# Splenic Sequestration Induced by Parvovirus B19: A Case Report

**DOI:** 10.7759/cureus.60937

**Published:** 2024-05-23

**Authors:** Shamsa Waleed, Maryam Aldabsa, Saria Gouher

**Affiliations:** 1 Pediatrics, Dubai Health Authority, Dubai, ARE; 2 Internal Medicine, American Hospital Dubai, Dubai, ARE

**Keywords:** thrombocytopenia, splenomegaly, parvovirus-b19, sickle cell disease (scd), acute splenic sequestration crises (assc)

## Abstract

Splenic sequestration crisis is a life-threatening complication of sickle cell disease (SCD), characterized by a sudden and huge accumulation of blood in the spleen, leading to rapid enlargement and may lead to organ failure. This case report discusses an unusual case of a splenic sequestration crisis in an adult with SCD. The patient's age, Parvovirus B19 infection, and concurrent retrocardiac pneumonia are all things that differentiate this case from our usual presentation. We will be discussing the clinical presentation, diagnostic methods, and management.

## Introduction

Sickle cell disease (SCD) presents a number of complications, among which acute splenic sequestration crisis (ASSC) and vasoocclusive crisis stand as primary life-threatening complications [1]. Typically considered mutually exclusive due to distinct clinical features, their concurrent manifestation is exceedingly rare but has been documented in isolated cases with considerable risk of fatal outcomes.

Vasoocclusive crisis arises from transient erythrocyte sickling with a marked reduction in deformability, which leads to increased adhesion to endothelial cells, rarely attributed to parvovirus B19 infection [2]. In contrast, acute splenic sequestration involves rapid hemoglobin loss (~2 g/dL) accompanied by splenomegaly and reticulocytosis commonly seen along with aplastic crisis, predominantly affecting pediatric patients with sickle cell anemia [3].

In this case report we present a 31-year-old male who had splenic sequestration induced by parvovirus B19.

## Case presentation

We present our patient, a 31-year-old male, with a significant past medical history of SCD. He presented to the hospital with complaints of back and generalized body pain, which limited his motion. His past medical history is remarkable for SCD and a recent admission due to pneumonia two months prior, which required hospitalization for one day, and after that, he was treated for it. Moreover, the patient usually has mild to moderate pain crisis, which was controlled at home with NSAIDs and rarely needed admission. On average, he gets admitted to the hospital once a year due to a sickle cell crisis. The record of the patient's family history reveals a genetic predisposition for this disease, as his father was identified to be an SCD carrier.

On admission, a respiratory rate of 18 breaths per minute was noted. The patient had an oxygen saturation of 100% on room air. He had no fever, as reviewed by a measurement of the tympanic body temperature, indicating that his reading was 36.7° centigrade. His peripheral pulse rate was 90 beats per minute, and the blood pressure reading was measured to be 131/81 mmHg. His height was 175 cm, and his weight was 70 kg.

Upon examination, the airway was clear. A cardiovascular examination showed no chest pain or tachycardia. Neurologically, he was alert and oriented, and the power of limbs was 5/5 with unremarkable neurological defect. Labs on admission showed a white blood cell (WBC) count of 3 million/mm^3^, red blood cell (RBC) count of 2.70 million/mm^3^, hemoglobin of 86.0 g/dL, reticulocytes of 0.1%, platelets of 97 mcL, and mean cell volume (MCV) of 90 fL (Table 1). Initial therapy included patient-controlled analgesic (morphine) 2 mg, intravenous hydration of normal saline rate of 125 mL/hour, and administration of hydroxyurea and tizanidine.

**Table 1:** Lab results WBC, white blood cell; RBC, red blood cell; MCV, mean cell volume

Test	Result on admission	Result on day 6	Normal values
WBC	3 million/mm^3^	2.0 million/mm^3^	4.5-11 million/mm^3^
RBC	2.70 million/mm^3^	1.96 million/mm^3^	4.5-6 million/mm^3^
Hematocrit	24.3%	17.4%	42-50%
Hemoglobin	86 g/dL	61 g/dL	13.5-18 g/dL
Platelets	97 thousand/mcL	45 thousand/mcL	150-450 thousand/mcL
Reticulocyte percentage	0.1%	2.7%	0.5-2.5%
MCV	90 fL	87 fL	83-101 fL

After a day of his admission, he started to have a fever, which was 38.7° centigrade (tympanic temperature) for three continuous readings. In addition to that on physical examination, the patient had severe left upper quadrant pain with a tender spleen on palpation. On day 6, blood pressure was 136/79 mmHg. Labs showed WBCs count of 2.0 million/mm^3^, RBCs count of 1.96 million/mm^3^, hemoglobin of 61 g/dL, reticulocytosis with a high reticulocyte percentage of 2.7%, and MCV of 87 fL (Table 1). This presentation and results go with the criteria of splenic sequestration.

**Figure 1:**
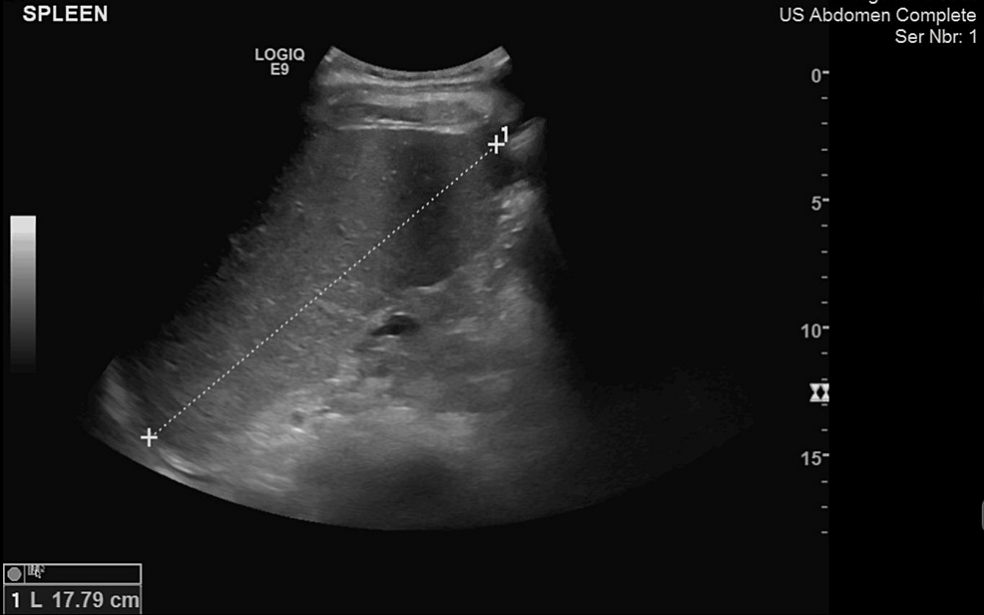
Ultrasound showing an enlarged spleen measuring 17.7 cm in length and normal homogenous echo-texture

**Figure 2:**
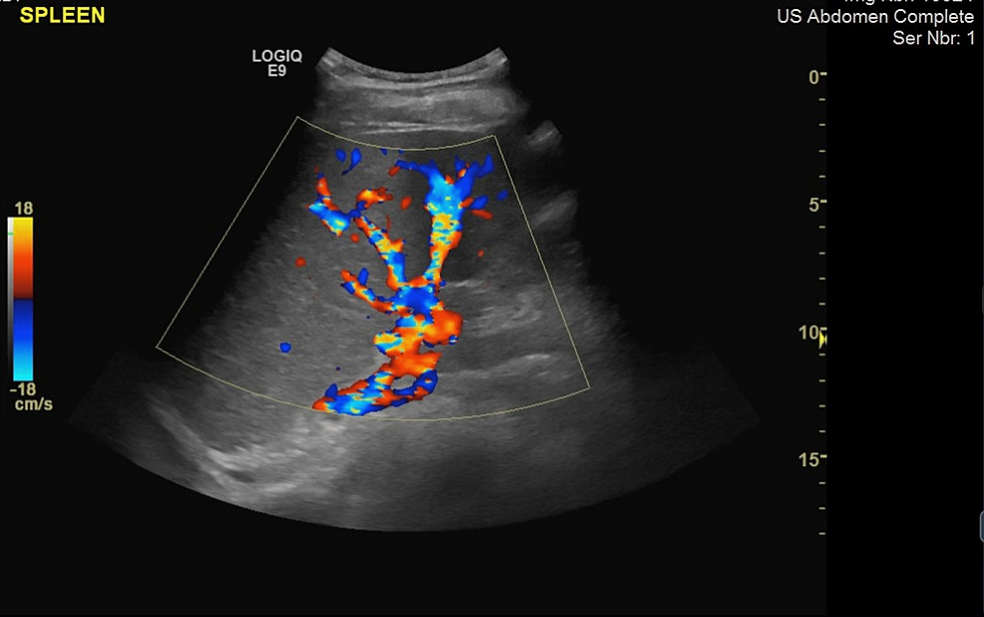
Ultrasound showing a fairly severe splenomegaly without any focal lesions

**Figure 3:**
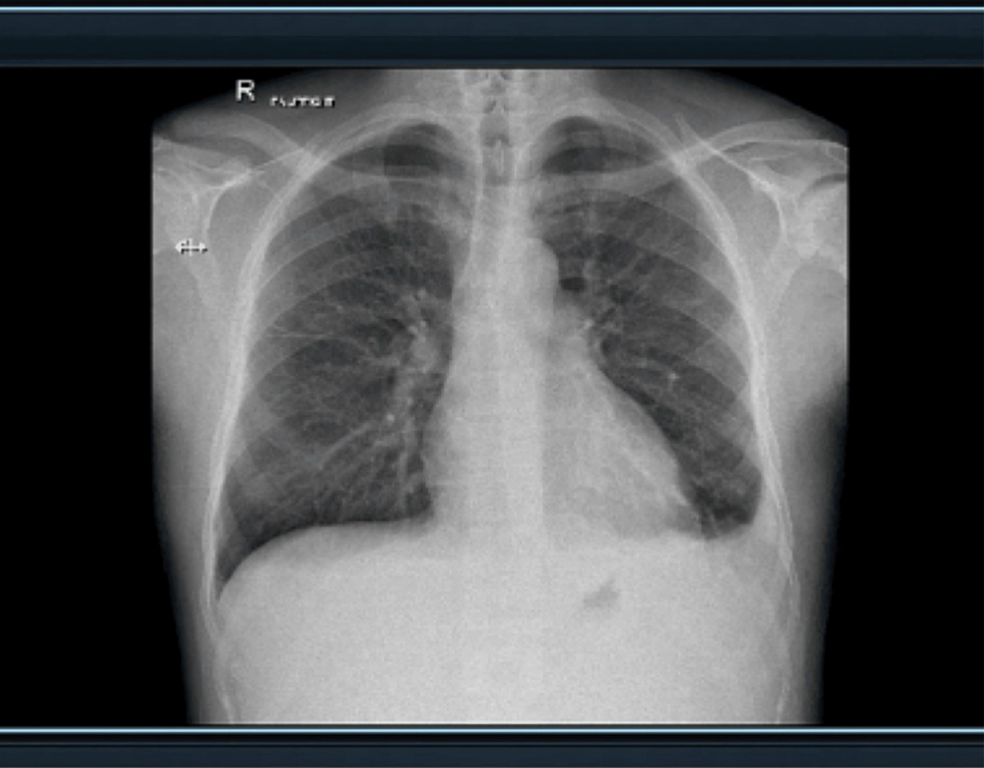
Chest X-ray showing clear lungs with no active or significant pathology. No hyperinflation or pneumothorax and no free subphrenic gas

The sonographer's findings from ultrasound showed (Figures 1, 2) that the patient's spleen measured 17.7 cm in length and had homogenous echotexture, which indicates an enlargement. A bilateral pleural effusion was noted. The findings were also consistent with splenic sequestration. A chest X-ray was done and the result was negative (Figure 3). Laboratory tests were then ordered for the patient, and the patient tested positive for Parvovirus B19 (Table 2). Although the patient had a known case of HbS homozygous, rapid sickle cell testing was done for confirmation and it came back positive (Table 3).

**Table 2:** Parvovirus B19 Ab, IgG, IgM, and S Results suggest recent infection.

Test	Result	Reference value
Parvovirus B19 Ab, IgG, S	Positive	Negative
Parvovirus B19 Ab, IgM, S	Positive	Negative

**Table 3:** Rapid sickle screen test The rapid sickle screen test is positive. It shows the presence of Hgb S that indicates the presence of SCD, an increase in Hgb F, and a decrease in both Hgb A and A2. SCD, sickle cell disease

Hemoglobin type	Results	Normal value
Hgb A	0%	96.7-97.8%
Hgb A2	1%	2.2-3.2%
Hgb F	41.4%	0-0.5%
Hgb S	57.6%	0%

In the beginning, the hemoglobin dropped 2 g/dL below the patient’s baseline so we transfused him with 2 g/dL of hemoglobin for the period of two days. After the platelet count dropped and the patient started having hemolysis, intravenous immunoglobulin (IVIG) was given at the rate of 0.4 g/kg daily for five days. In addition to that, methylprednisolone 500 mg was added. Ceftriaxone, an empiric antibiotic, was started as a prophylaxis on day 1. Since the patient was a visiting patient, follow-up was done through telemedicine appointment; as he mentioned, he was doing better with good health, and the pain resolved completely with no complications.

## Discussion

This is a unique case of a splenic sequestration crisis in an adult with SCD. The patient's age, Parvovirus B19 infection, and past medical history of pneumonia two months ago make this case unique in comparison with the previously reported cases of a 38-year-old woman, and of a 23-year-old female patient who progressed to death [4,5]. Parvovirus B19 viral infection is commonly associated with splenic sequestration in SCD patients in children below 15 years old and it is rarely presented in adults [6]. Parvovirus B19 increases the severity of the condition by destroying the erythroid precursor cells and leads to decreased production of RBCs [7]. Splenic sequestration is diagnosed based on features such as anemia, thrombocytopenia, life-threatening rapid spleen enlargement, sudden weakness, hypotension, volume depletion, and tachycardia [6]. These features resemble those of hypovolemic shock [6]. Research shows that splenic sequestration crises have the highest frequency in children under 15 years old and are diagnosed with sickle cell anemia. The condition affects an average of 12% of children [6]. The condition leads to about 60% splenomegaly in children. The decision to initiate a transfusion, correct anemia, and administer IVIG was driven by the need to counteract the effects of Parvovirus B19 and alleviate symptoms [6]. In a study done by Teikyo University School of Medicine, Tokyo, Japan, it was shown that patients who have prominent platelet sequestration in the spleen responded to IVIG, and it decreased by 20-30% [8]. This supports the decision to administer IVIG. The importance of monitoring hemoglobin and reticulocyte count became evident in assessing the response to treatment, and the possibility of splenectomy was considered a preventive measure. 

## Conclusions

In conclusion, this case report unfolds a fascinating narrative of an SCD patient presenting with splenic sequestration induced by Parvovirus B19. The interaction between genetic predisposition and viral infection adds layers of complexity to the clinical scenario. The case serves as a reminder to healthcare providers that while sickle cell crises often follow predictable patterns, occasional deviations can lead to unforeseen complications. The management decisions made in this case, including transfusion, IVIG administration, and consideration of splenectomy, demonstrate the importance of a multidisciplinary approach in addressing such complex scenarios. 
